# Cardiac Mean Electrical Axis in Thoroughbreds—Standardization by the Dubois Lead Positioning System

**DOI:** 10.1371/journal.pone.0169619

**Published:** 2017-01-17

**Authors:** Cássia Fré da Costa, Nelson Samesima, Carlos Alberto Pastore

**Affiliations:** Clinical Unit of Electrocardiography, Heart Institute (InCor), Hospital das Clínicas da Faculdade de Medicina, Universidade de São Paulo, São Paulo, Brazil; University of Minnesota, UNITED STATES

## Abstract

**Background:**

Different methodologies for electrocardiographic acquisition in horses have been used since the first ECG recordings in equines were reported early in the last century. This study aimed to determine the best ECG electrodes positioning method and the most reliable calculation of mean cardiac axis (MEA) in equines.

**Materials and Methods:**

We evaluated the electrocardiographic profile of 53 clinically healthy Thoroughbreds, 38 males and 15 females, with ages ranging 2–7 years old, all reared at the São Paulo Jockey Club, in Brazil. Two ECG tracings were recorded from each animal, one using the Dubois lead positioning system, the second using the base-apex method. QRS complex amplitudes were analyzed to obtain MEA values in the frontal plane for each of the two electrode positioning methods mentioned above, using two calculation approaches, the first by Tilley tables and the second by trigonometric calculation. Results were compared between the two methods.

**Results:**

There was significant difference in cardiac axis values: MEA obtained by the Tilley tables was +135.1° ± 90.9° *vs*. -81.1° ± 3.6° (p<0.0001), and by trigonometric calculation it was -15.0° ± 11.3° *vs*. -79.9° ± 7.4° (p<0.0001), base-apex and Dubois, respectively. Furthermore, Dubois method presented small range of variation without statistical or clinical difference by either calculation mode, while there was a wide variation in the base-apex method.

**Conclusion:**

Dubois improved centralization of the Thoroughbreds' hearts, engendering what seems to be the real frontal plane. By either calculation mode, it was the most reliable methodology to obtain cardiac mean electrical axis in equines.

## Introduction

The Thoroughbred racing horse is a widespread breed in racetracks around the world. Thoroughbreds’ physical constitution favors the achievement of a greater speed, and for this reason they take part in many other sports modalities, such as Polo, and are used in cross-breeding with equines as the Quarter Horse to enhance the speed of these animals in their usual activities.

Studies using the electrocardiogram (ECG) in equines were first reported in 1912 by Thomas Lewis [1]. Since that time the ECG proved itself as a painless, inexpensive tool [2–3], and the gold standard for detection and characterization of arrhythmias in horses [4]. In addition to breed, other details such as the animals’ age, gender and physical conditioning may influence the ECG findings [3,5].

The results obtained from ECG recordings are significantly altered depending on the acquisition method that is used. In the literature we can find reports presenting many ways of positioning the electrodes on the animals. The purpose of ECG systems is to record waveforms and complexes that evaluate the cardiac electrical conduction process and collect data concerning the direction and magnitude of the cardiac vector [6]. In this study we compared two different lead positioning techniques, the Dubois [7] and the base-apex [5,8] methodologies.

The mean electrical axis (MEA) reveals the direction and orientation of ventricular depolarization. Even though MEA can be measured in any plane, its characterization for ventricular evaluation uses mainly the frontal plane. The main objective of determining the mean electrical axis is to establish criteria for ventricular dilatation or hypertrophy, and to detect intraventricular conduction defects [9]. The electrical axis can also express the status of the heart relative to the efforts required by the type of training and sports modality usually endured by the animals [8]. For all these purposes, it is assumed that the heart lies in the center of a triangle, as established by Einthoven [6,10].

The objective of this study was to compare results of the mean electrical axis obtained in Thoroughbreds using the two methods mentioned above, base-apex and Dubois, and to find out which of the two MEA calculation methods, Tilley’s or trigonometric, is the most appropriate for equines.

## Materials and Methods

### Study subjects

Fifty-three Thoroughbred equines, 38 males and 15 females, with ages ranging 2 to 7 years old, all reared at the São Paulo Jockey Club, were evaluated. Details on the animals’ husbandry were obtained in the website http://www.studbook.com.br/solicita_nome_animal_campanha.htm (the Brazilian Stud Book) [11]. They are fed with commercial concentrates and forages, and are kept in individual boxes. All the horses undergo daily individual training in the racetrack. The 2-year-old horses do not compete yet.

The exclusion criterion was the presence of any health disorder, including cardiopathy, which was ruled out by clinical examination.

This study underwent ethical review and was given approval by the Institutional Review Board of the Heart Institute (InCor) of the University of São Paulo Medical School Hospital. The care and use of the equines complied with local animal welfare laws, guidelines and policies and was approved by the São Paulo Jockey Club board of directors.

### Electrocardiogram

Electrocardiographic recordings were acquired in quiet environment, with a rubber mat placed on the floor of the stall for electrical insulation. The horses stood in orthostatic position with parallel limbs, and no chemical constraint was used.

A portable TEB ECGPC VET 12-channel-electrocardiograph (TEB—Tecnologia Eletrônica Brasileira, São Paulo, Brazil) was used. The device was set at 25 mm/sec paper speed and sensitivity of 1cm = 1mV. Bipolar (DI, DII, DIII), augmented unipolar (aVR, aVL, aVF) and precordial (rV2, V2, V4 and V10) leads were recorded.

### Leads

The electrodes were fixed to alligator metal clips, which were attached to the horse’s skin after being moistened with alcohol.

**Fig 1:**
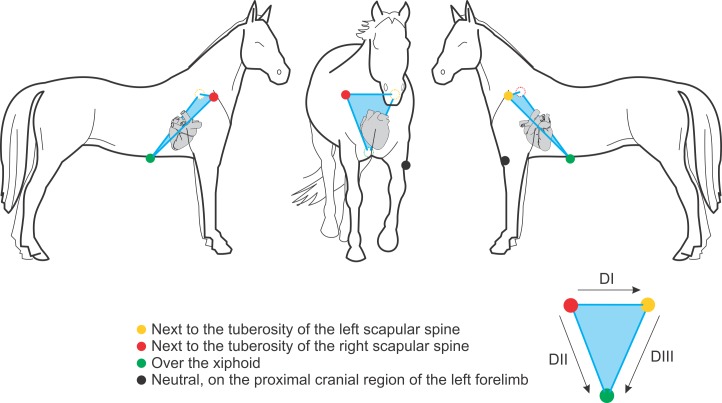
Disposition of electrodes by the Dubois method. The illustration depicts the disposition of electrodes by the Dubois method.

Two electrocardiograms were obtained in sequence from each horse. Dubois method was used first, by placing the yellow and the red electrodes respectively next to the spine tuberosity of the left and right scapulae; the green electrode was placed on the xiphoid process of the sternum, and the black electrode on the proximal cranial region of the left forelimb (Fig 1).

**Fig 2:**
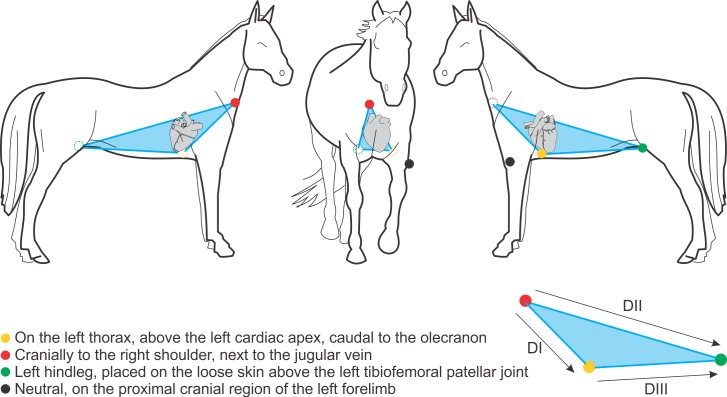
Base-apex electrodes positioning. The illustration depicts the disposition of electrodes by the base-apex method.

The second ECG, using the base-apex method, consists in placing the yellow electrode above the left cardiac apex, caudal to the olecranon; the red electrode, cranially to the right shoulder, next to the jugular vein; the green electrode, above the left tibiofemoral patellar joint, and the black electrode on the proximal cranial region of the left forelimb (Fig 2).

### Electrocardiographic variables

Parameters for evaluation of the mean electrical axis were obtained by systematic analysis of the QRS complex amplitude values in millivolts (mV), which were semi-automatically measured in leads DI, DIII and aVF from the inbuilt electrocardiograph software, by a sole observer (first author). P-wave duration, PR interval and QRS duration and amplitude in all leads were also semi-automatically measured.

### Calculation of the mean electrical axis

Values of the QRS complex angles were thus obtained: first, using pre-defined tables specially developed for cats and dogs, with QRS complex amplitudes measured in leads DI and DIII, according to Tilley’s description (1992) [9]; second, from trigonometric calculations (α tangent) of QRS complex amplitudes in leads DI and aVF, the same used in human medicine.

#### Trigonometric calculation

The Einthoven triangle is based upon bipolar leads that are represented by six leads as: DI, DII, DIII, aVR, aVL, aVF, all of them equally distributed within 30 degrees. Using the trigonometry concept for obtaining angles from a right triangle, there are three ways to calculate the desired axis: sine, cosine and tangent. DI and aVF are orthogonal leads and they are used to calculate the axis of any wave. The QRS amplitudes in DI and aVF are the known measurements in every ECG. Thus, to obtain the QRS mean electrical axis it is necessary to use the tangent formula from trigonometry that is obtained by dividing the opposing cathet by the adjacent cathet. (opposing cathet is aVF; adjacent cathet is DI).

The two calculation modes were applied to the two methods of ECG acquisition.

### Statistical analysis

Continual variables were expressed as mean ± standard deviation values; categorical variables in per cent values. Paired *t*-test and Fisher’s test were used to compare groups; significance level was set to be lower than or equal to 5%.

## Results

Table 1 displays the horse’s weights, heights and ages. All equines underwent a previous clinical examination.

**Table 1:** Demographic characteristics (mean ± standard deviation) of animals undergoing electrocardiographic examination.

	Age (years)	Weight (kg)	Height (m)
**Mean ± SD**	4.0 ± 1.3	474 ± 32	1.62 ± 0.04
**Median**	4.0	472	1.63
**Min.**	2.0	416	1.54
**Max.**	7.0	545	1.70
**Min. 95% C.I.**	1.0	456	1.53
**Max. 95% C.I.**	5.0	463	1.71

**S.D.** = standard deviation; **C.I**. = confidence interval; **Kg** = kilograms; **m** = meters

**max** = maximum; **min** = minimum.

For easier visualization of mean electrical axis results we divided ECGs in two groups; A, obtained by the base-apex, and B, by the Dubois leads positioning system.

In a further division, the values obtained from leads DI and DIII, calculated according to Tilley tables, composed subgroups A1 and B1. The trigonometric calculations obtained from QRS amplitudes in leads DI and aVF, similar to those measured for humans, composed subgroups A2 and B2.

Mean Cardiac Axis in the ECGs of group A (base-apex), as calculated by Tilley (A1), was +135.1° ± 90.9°, while MEA trigonometrically calculated (A2) was -15.0° ± 11.3°, p< 0.0001.

**Fig 3:**
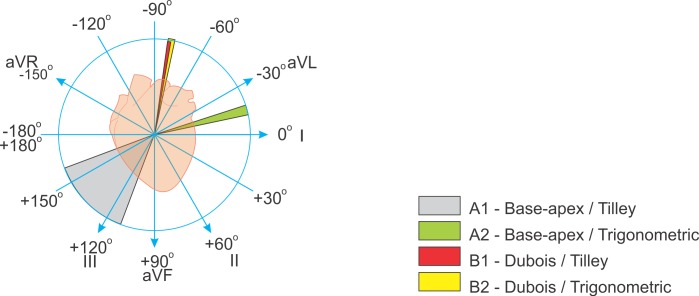
Graphic representation of the cardiac axis. Cardiac axes measured in Thoroughbreds from ECGs acquired by Dubois and base-apex methods, by Tilley tables and trigonometric calculations, with range of variation expressed in degrees.

In the ECGs of group B (by Dubois), Tilley’s method (B1) resulted in mean -81.1° ± 3.6°, while trigonometric calculation (B2) yielded mean -79.9° ± 7.4°, p = 0.3128. Fig 3 displays and compares all the above results.

A significant difference in cardiac axis values was evidenced by comparing the base-apex and the Dubois methods, both using the Tilley tables and by trigonometric calculation. The mean angle obtained by Tilley tables was +135.1° ± 90.9° versus -81.1° ± 3.6°; and by trigonometric calculation it was -15.0° ± 11.3° versus -79.9° ± 7.4° (base-apex and Dubois, respectively; p<0.0001 for both).

Additionally, the base-apex lead positioning method yielded a great variation of axis values by the two angle calculation methodologies, with even greater variation for those from Tilley tables. On the other hand, results obtained by the Dubois method presented a small range of variation without statistical or clinical difference, by either calculation method (Table 2).

**Table 2:** Comparison of cardiac axis values obtained with the base-apex and the Dubois lead positioning methods, calculated according to Tilley (A1 and B1) and by trigonometric calculation (A2 and B2).

	A1	A2	B1	B2
**Mean ± S.D.**	+135.1 ± 90.9	-15.0 ± 11.3	-81.1 ± 3.6	-79.9 ± 7.4
**Min.**	-173	-35.8	-90	-86.6
**Max.**	134	+17.1	-75	-38.4
**Median**	161.0	-14.0	-81.0	-81.1
**Min. 95% C.I.**	110.6	-18.0	-82.1	-81.9
**Max. 95% C.I.**	159.5	-12.0	-80.1	-77.9

**C.I. =** confidence interval; **max =** maximum; **min =** minimum; **SD =** standard deviation.

Complete data used for calculation of the above results can be found in S1 Table.

## Discussion

In the present study we sought to establish which of two methodologies used to calculate the mean electrical axis in horses was more precise. The first, described by Tilley [9], consists of four tables designed for cats and dogs, using leads DI and DIII. The second method, which is trigonometrically calculated using leads DI and aVF, is broadly used in human medicine around the world. As discussed below, our results showed that either approach can be used for equines, just as long as the electrocardiographic tracings are properly acquired.

Tilley’s values of MEA were +135.1° ± 90.9° versus -81.1° ± 3.6° while MEA trigonometrically calculated was -15.0° ± 11.3° versus -79.9° ± 7.4°, using base-apex and Dubois, respectively. These data are very similar to the ones obtained by Colahan and cols. (1991) [12], performing vectorcardiograms in equines. Results shown in their transverse and sagittal planes exhibited the same QRS axis direction and orientation as those found in our study.

The results of this study show a huge difference between values from the two ECG acquisitions. This calls for imperative standardization of technique. The two sets of measurements simply cannot be compared.

Another interesting fact is the large difference in MEA values achieved through the base-apex ECG acquisition, either by Tilley or trigonometric calculation. In our view, this acquisition approach uses inappropriate sites of electrode placement. Additionally, those values do not reflect the real information about what is known so far concerning the anatomical electric activation of the Purkinje fibers in horses. This difference was not found when comparing the results from the Dubois methodology, thus showing what looks like a more appropriate electrocardiographic acquisition.

Our findings using Dubois electrodes placement clearly demonstrate that the mean electrical axis in Thoroughbreds is located on the left, cranially and dorsally, in 100% of the study population.

To explain this particular ECG finding, we based our understanding on the anatomical description of a sheath of connective tissue which involves the electrical conduction system of ungulates from the septum until the apical region [13–14]. This means that the equine conduction system is electrically insulated up to its insertion into the ventricular apical region, which implies that their ventricular activation runs from the apex towards the basal region [15].

This unique characteristic of their cardiac electrical system anatomy can explain this left and upwardly QRS orientation. The animals of this study are elite athletes, therefore the absence of cardiovascular diseases on clinical examination, allied to their great performance, ensures that this pattern is normal, rather than pathological trait. Also, we excluded the variable gender as cause of axis deviation.

We are aware that many authors support the theory of vector cancellation, established in the middle of last century [16,17] based on mathematical models constructed from the initial studies by Frank (1955), which represented the heart as a single dipole, variable in strength and axial orientation, but constant in location. In our view, the anatomy of the equine electrical conduction system above described can explain our animals’ peculiar cardiac activation.

The electrocardiogram is a tool used to access the electrical activity of the heart as a whole, which permits the evaluation of the entire electrical activity of the heart. This means that the method applied should be able to evaluate the cardiac rhythm, heart rate, mean P, QRS and T axes, the atrioventricular conduction of impulses, any enlargement of the heart chambers (both atria and ventricles), as well as the ventricular repolarization.

As explained above, equine ECG findings are restricted to the use of only a few variables, such as heart rhythm and heart rate. The fact that certain variables provided by the ECG are no longer used (such as the mean electrical axis) is no proof that they are not valuable. On the contrary, they are regarded as useless information because they have been mistakenly obtained, and consequently they cannot provide the expected correlations with the heart physiology.

Some authors compare their results with other results presented in papers in which different ECG acquisition methods were used; and even other breeds [15,18]. Based on the findings of this study, we think this just can’t be done.

The literature describes different methods to obtain electrocardiographic recordings in horses, which place the ECG electrodes in different locations of their bodies (Dubois, base-apex, Einthoven and other modified ones). This last category has been used by some authors just to check for the presence and characterization of arrhythmias, not taking advantage of all the ECG potential for cardiovascular information [19,20].

The conclusion of the present study (MEA evaluation) is that Dubois is currently the most reliable method for the correct acquisition, interpretation and correlation between electrocardiographic findings and any possible electrical/mechanical cardiac disorders in Thoroughbreds. To achieve our objective we compared two different methods, Dubois and base-apex, mainly to bring up how and why one is better than the other. Besides, to enhance our theory, some discussion concerning the Einthoven’s method was taken into account as well. As mentioned above, although it is possible to obtain the angles in the three orthogonal planes, we are aware that only those obtained in the frontal plane portend any electrocardiographic meaning.

This notion is also shared by Ayala et al. (2000) [7], who mentioned the Einthoven´s method as inadequate for equines due to the influence of limb position, in addition to the inappropriate electrodes positioning relative to the location of the heart in the chest.

Authors who used the base-apex method ascribed their choice to the more practical positioning of electrodes provided by this system, in addition to the easier reading of tracings due to the regularity and large amplitude displayed by QRS complexes [8, 21]. In our experience with Thoroughbreds, this method was not the best choice, since the animals felt uncomfortable, especially with the green electrode attachment, what often provoked backwards kicking.

Our results show that the angles obtained by the base-apex method lack any meaning; however, they compare with those presented by Dumont et al (2011) [21] in their study of Arabian horses (+135.1° ± 90.9° *vs*. +105.0° ± 7.8°).

Even nowadays some authors [19] consider Einthoven as a reference method for horse ECG. The problem here is that this method provides the transverse plane, which can only be used to express heart rate and heart rhythm, whilst the frontal plane cannot be thus evaluated.

**Fig 4:**
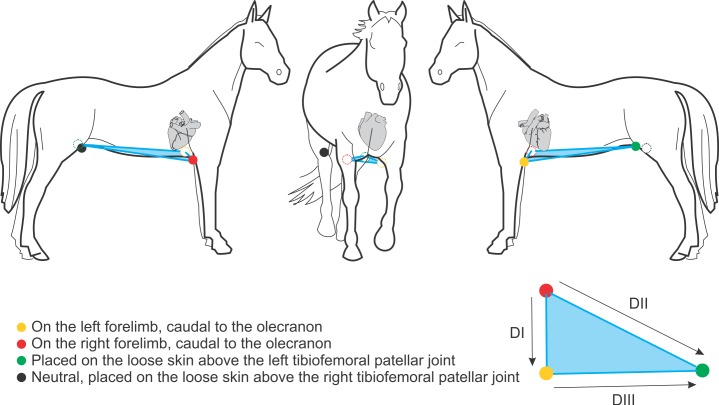
Disposition of electrodes by the Einthoven method. The illustration depicts the disposition of electrodes by the Einthoven’s method.

Other authors reported results of QRS angles obtained using Einthoven’s method, which ranged from -119° to +120° [2]; or else, from +59.4° ± 25.56° to 68.74° ± 33.02° [15]. Again we are faced with another conceptual mistake. Once more, it is important to visualize where the red, yellow and green electrodes are positioned. In this method, their disposition, which allegedly represented the frontal plane, in fact represent the transverse plane (Fig 4). Thus, the information about the position of electric phenomena by the Einthoven’s method reflect what actually occurs on the right side, the left side, cranially (anterior) and caudally (posterior).

Those, such as Ayala et al. [7] who chose the Dubois leads system, reported this as the most adequate for equines, since the lead positioning has a great influence on the centralization of the heart in the thorax. Also, in our experience the place of electrodes attachment in the horse is safer for the technicians.

It is extremely important to remember that DI, DII and DIII leads are obtained respectively from the red and yellow electrodes, the red and green electrodes, and the yellow and green electrodes. Thus, the main difference between these two methodologies (Dubois vs. base-apex) is clearly exposed when we compare Figs 1 and 2. It is consensus among authors that the electrode positioning of the Dubois leads system (Fig 1) provides an actual frontal plane; it implies a possibility of truly displaying the electric phenomena located on the right, on the left, dorsally (superiorly) and ventrally (inferiorly). In the base-apex method (Fig 2), the red electrode is displaced from the crest of the right scapula to the base of the right jugular vein sulcus, and the yellow electrode is displaced from the crest of the left scapula to the cardiac apex. Consequently, the leads that represented the frontal plane now become representatives of the sagittal plane. Therefore, the localization of electric phenomena by the base-apex method is now represented by the dorsal (superior), ventral (inferior), cranial and caudal positions. Thus, even if any electrocardiogram waveform (P, QRS, T) angle can be obtained by this method, it is not adequate, since by electrocardiographic definition the angles of any ECG waveforms must be calculated from the frontal plane [10,22].

All the above cited papers have shown definitely distinct cardiac axis values between equine breeds, and even within the same breed, which calls for imperative standardization of values for specific breeds [15]. Nevertheless, before we standardize axis values for different breeds, there is a crucial need to standardize the methodology used to acquire the ECG tracings.

**Fig 5:**
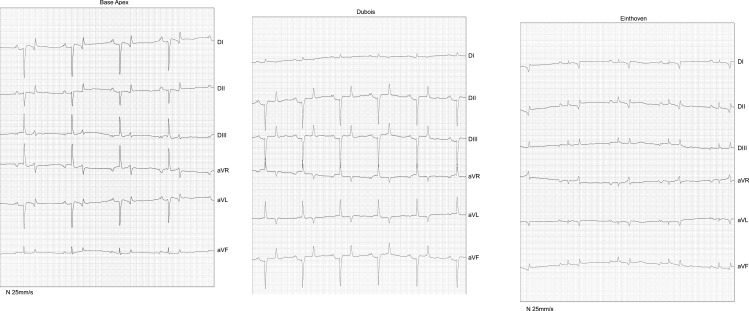
**Tracings with the three electrode positioning methods: Panel A. base-apex; Panel B. Dubois; and Panel C. Einthoven.** Three ECG tracings illustrating the different amplitudes and directions of mean electrical axes obtained with the different electrode positioning methods.

In the present study we demonstrated that the different disposition of electrodes by the Dubois, base-apex and Einthoven methodologies directly and significantly influences the results (Fig 5). Its change reflects a complete modification of the spatial position of what is inferred as the frontal plane. Figs 1, 2 and 4 clearly display that the Dubois, base-apex and Einthoven methodologies provide electrocardiographic data respectively about the animal’s frontal, sagittal and transverse planes. All the textbooks on electrocardiography (e.g. Bayés de Luna A, 2008; Grauer K, 1998) [10,22] unanimously agree on the form of obtaining any waveform angle, and that this must be done with data from the frontal plane.

These three tracings show us some interesting features. It is generally agreed that the left ventricle is predominant over the right ventricle, because of its greater mass and diameter, in all the mammals. Thus, when we examine the mean cardiac axis of any mammal we should expect to notice its leftward dislocation. However, analyzing the tracings obtained by the base-apex and the Einthoven methods, we clearly notice that the MEA is inexplicably dislocated to the right side. The tracing generated by the Dubois electrode positioning, on the other hand, shows the MEA located according to our expectations.

Furthermore, ventricular activation in the Einthoven and base-apex electrode positioning appears as if it was processed from top to bottom, which is not consistent with the explanation provided some paragraphs above [13] about the direction of ventricular activation in equines (bottom-up). Only the Dubois method shows both the correct activation orientation/direction (the bottom-up ventricular activation, in addition to leftward MEA dislocation).

The aim of using equines kept at the São Paulo Jockey Club was to demonstrate the electrocardiographic cardiac behavior of healthy, high performance racehorses.

The significance of the present study lies in the standardization of cardiac axis values for Thoroughbreds, since some very significant differences are found between the many breeds of this species.

## Conclusion

Thoroughbreds present a cranially, dorsally and leftward oriented cardiac axis, as a normal physiologic feature.

This study of mean electrical axis demonstrates that the Dubois leads system is currently the most reliable method to be employed for recording electrocardiograms in Thoroughbreds. The Dubois leads positioning also allows veterinarians to use either MEA calculation method, Tilley’s or trigonometric.

## Limitation

Although the Dubois ECG methodology presented more reliable results in our study, we observed an important limitation regarding the positioning of electrodes. The values obtained sometimes are in disagreement with the equine heart anatomy. Therefore, more studies into this subject are required.

## Supporting Information

S1 Table Raw data on Thoroughbreds for mean cardiac axis calculation. S1 Table contains the amplitude values of QRS complex in leads DI, DIII and aVF collected from all the 53 Thoroughbred horses using Tilley tables and trigonometric calculation of mean cardiac axis by the two different lead positioning methods.
(PDF)
