# Supplementary material for: Cardiac Mean Electrical Axis in Thoroughbreds—Standardization by the Dubois Lead Positioning System
Source: PLoS One. 2017 Jan 17;12(1):e0169619. doi: 10.1371/journal.pone.0169619 (PMC5241011; doi:10.1371/journal.pone.0169619)
Supplement: S1 Table — contains the amplitude values of QRS complex in leads DI, DIII and aVF collected from all the 53 Thoroughbred horses using Tilley tables and trigonometric calculation of mean cardiac axis by the two different lead positioning methods. (PDF) [file pone.0169619.s001.pdf]

Horse	VOLTAGE						B1	B2	A1	A2
	DI		DIII		aVF		Tilley	Trigonometric	Tilley	Trigonometric
	Dubois	Base-apex	Dubois	Base-apex	Dubois	Base-apex	Dubois	Dubois	Base-apex	Base-apex
1	0,3	-3,1	-2,1	2,7	-1,9	1,4	-82,0	-80,6	159,0	-23,5
2	0,2	-3,4	-2,9	1,9	-2,7	0,4	-90,0	-84,9	175,0	-6,3
3	0,3	-2,9	-2,2	2,7	-2,0	1,6	-76,0	-81,7	159,0	-29,2
4	0,5	-1,4	-2,0	0,8	-1,8	0,1	-76,0	-75,5	175,0	-3,3
5	0,4	-2,6	-1,6	2,2	-1,4	1,1	-75,0	-71,8	161,0	-23,4
6	0,5	-2,9	-2,8	2,1	-2,5	1,0	-81,0	-78,1	169,0	-19,5
7	0,3	-2,7	-2,2	2,0	-2,0	0,7	-82,0	-80,0	161,0	-14,2
8	0,3	-3,5	-2,8	2,3	-2,6	0,8	-81,0	-84,1	166,0	-13,0
9	0,4	-2,4	-3,2	0,8	-3,0	0,3	-81,0	-81,8	-173,0	-7,7
10	0,3	-2,7	-2,7	0,5	-2,7	0,5	-79,0	-84,0	-161,0	-11,2
11	0,2	-3,2	-2,7	2,2	-2,8	0,6	-90,0	-85,5	169,0	-10,6
12	0,4	-3,9	-3,1	3,2	-3,0	1,4	-81,0	-81,9	164,0	-19,8
13	0,4	-1,7	-2,5	1,2	-2,3	0,6	-79,0	-80,7	161,0	-18,3
14	0,3	-2,6	-2,7	1,8	-2,6	0,6	-79,0	-83,8	161,0	-12,6
15	0,3	-2,7	-1,7	2,6	-1,5	1,6	-79,0	-79,1	150,0	-31,0
16	0,5	0,8	-1,3	-0,6	-0,9	-0,3	-77,0	-60,2	-16,0	-21,6
17	0,4	-1,8	-2,3	1,2	-2,1	0,5	-79,0	-80,7	173,0	-15,4
18	0,4	-2,2	-2,3	1,7	-2,1	0,7	-79,0	-78,2	164,0	-17,2
19	0,4	-1,1	-1,2	1,3	-1,0	0,8	-76,0	-67,3	142,0	-35,8
20	0,3	-3,8	-3,0	2,2	-2,9	0,3	-81,0	-84,4	180,0	-4,1
21	0,3	-1,7	-2,2	1,6	-2,0	0,9	-82,0	-83,0	150,0	-26,7
22	0,5	-2,5	-2,3	1,3	-2,0	0,1	-79,0	-77,4	173,0	-2,0
23	0,4	-2,1	-3,0	1,1	-2,7	-0,1	-81,0	-81,1	180,0	2,1
24	0,3	-3,5	-2,8	2,8	-2,6	1,1	-81,0	-84,0	158,0	-17,0
25	0,4	-2,2	-2,6	1,1	-2,4	0,2	-79,0	-81,0	180,0	-4,2
26	0,5	-2,2	-2,1	1,7	-1,9	0,6	-76,0	-76,0	164,0	-14,0
27	0,3	-3,2	-2,8	2,6	-2,7	1,1	-81,0	-84,3	159,0	-19,1
28	0,2	-2,6	-2,1	1,9	-2,0	0,6	-90,0	-83,2	161,0	-12,3
29	0,3	-1,7	-1,7	1,6	-1,5	0,8	-79,0	-77,3	150,0	-25,9
30	0,2	-3,4	-2,2	2,4	-2,2	0,8	-85,0	-83,6	166,0	-12,9
31	0,2	-2,9	-2,5	1,7	-2,4	0,3	-90,0	-84,7	180,0	-5,0
32	0,4	-1,6	-2,6	1,0	-2,4	0,1	-79,0	-81,5	169,0	-5,0
33	0,1	-2,3	-1,8	1,8	-1,8	0,7	-87,0	-85,7	161,0	-17,5
34	0,3	-2,4	-2,2	1,7	-2,1	0,7	-82,0	-81,8	173,0	-15,4
35	0,3	-2,5	-2,5	1,7	-2,4	0,5	-79,0	-84,0	173,0	-11,6
36	0,3	-3,0	-2,0	2,3	-1,9	1,2	-82,0	-79,7	159,0	-22,7
37	0,3	-2,4	-2,9	1,5	-2,7	0,3	-81,0	-84,4	173,0	-5,9
38	0,4	-2,1	-2,2	1,8	-1,9	1,0	-79,0	-78,4	150,0	-24,6
39	0,3	-3,7	-2,7	2,2	-2,6	0,4	-79,0	-83,6	175,0	-6,8
40	0,4	-2,3	-2,4	1,2	-2,2	-0,1	-79,0	-80,4	-173,0	2,2
41	0,3	-3,5	-3,1	2,0	-2,9	0,1	-81,0	-83,4	175,0	-1,3
42	0,4	-2,2	-2,4	0,9	-2,2	0,0	-79,0	-81,0	180,0	0,0
43	0,2	-1,8	-1,5	1,8	-1,3	1,1	-83,0	-79,8	150,0	-30,5
44	0,2	-1,4	-1,2	1,5	-1,1	0,7	-81,0	-79,6	147,0	-26,7
45	0,1	-1,9	-1,7	1,4	-1,7	0,5	-87,0	-86,6	167,0	-13,7
46	0,3	-1,7	-1,6	2,0	-1,4	1,1	-79,0	-78,7	136,0	-32,4
47	0,3	-3,9	-3,2	2,6	-3,1	0,8	-81,0	-85,4	172,0	-11,1
48	0,3	-1,3	-2,2	1,2	-1,9	0,5	-82,0	-80,9	154,0	-23,2
49	0,3	-1,0	-1,8	0,1	-1,5	-0,3	-82,0	-79,5	-155,0	17,1
50	0,5	-0,5	-0,7	0,7	-0,4	0,5	-86,0	-38,4	134,0	-47,7
51	0,3	-1,9	-1,5	1,2	-1,3	0,4	-79,0	-78,5	173,0	-11,4
52	0,3	-2,8	-2,3	1,8	-2,2	0,5	-79,0	-82,0	169,0	-10,4
53	0,2	-0,9	-2,1	1,2	-2,0	0,4	-85,0	-83,0	136,0	-20,6

**Mean**	-81,1	-79,9	135,1	-15,0
**SD**	3,6	7,4	90,9	11,3
**Median**	-81,0	-81,1	161,0	-14,0
	**B1**	**B2**	**A1**	**A2**
